# Differential Wound Healing Capacity of Mesenchymal Stem Cell-Derived Exosomes Originated From Bone Marrow, Adipose Tissue and Umbilical Cord Under Serum- and Xeno-Free Condition

**DOI:** 10.3389/fmolb.2020.00119

**Published:** 2020-06-24

**Authors:** Diem Huong Hoang, Tu Dac Nguyen, Hoang-Phuong Nguyen, Xuan-Hung Nguyen, Phuong Thi Xuan Do, Van Duc Dang, Phuong Thi Minh Dam, Hue Thi Hong Bui, Mai Quynh Trinh, Duc Minh Vu, Nhung Thi My Hoang, Liem Nguyen Thanh, Uyen Thi Trang Than

**Affiliations:** ^1^Vinmec Research Institute of Stem Cell and Gene Technology (VRISG), Vinmec Health Care System, Hanoi, Vietnam; ^2^Vinmec Hightech Center, Vinmec Healthcare System, Hanoi, Vietnam; ^3^College of Health Sciences, Vin University, Hanoi, Vietnam; ^4^University of Science, Viet Nam University, Hanoi, Vietnam

**Keywords:** exosomes, mesenchymal stem cells, BMMSC-derived exosomes, ADMSC-derived exosomes, UCMSC-derived exosomes, growth factors, wound healing

## Abstract

Exosomes are nano-scale and closed membrane vesicles which are promising for therapeutic applications due to exosome-enclosed therapeutic molecules such as DNA, small RNAs, proteins and lipids. Recently, it has been demonstrated that mesenchymal stem cell (MSC)-derived exosomes have capacity to regulate many biological events associated with wound healing process, such as cell proliferation, cell migration and blood vessel formation. This study investigated the regenerative potentials for cutaneous tissue, in regard to growth factors associated with wound healing and skin cell proliferation and migration, by exosomes released from primary MSCs originated from bone marrow (BM), adipose tissue (AD), and umbilical cord (UC) under serum- and xeno-free condition. We found crucial wound healing-mediated growth factors, such as vascular endothelial growth factor A (VEGF-A), fibroblast growth factor 2 (FGF-2), hepatocyte growth factor (HGF), and platelet-derived growth factor BB (PDGF-BB) in exosomes derived from all three MSC sources. However, expression levels of these growth factors in exosomes were influenced by MSC origins, especially transforming growth factor beta (TGF-β) was only detected in UCMSC-derived exosomes. All exosomes released by three MSCs sources induced keratinocyte and fibroblast proliferation and migration; and, the induction of cell migration is a dependent manner with the higher dose of exosomes was used (20 μg), the faster migration rate was observed. Additionally, the influences of exosomes on cell proliferation and migration was associated with exosome origins and also target cells of exosomes that the greatest induction of primary dermal fibroblasts belongs to BMMSC-derived exosomes and keratinocytes belongs to UCMSC-derived exosomes. Data from this study indicated that BMMSCs and UCMSCs under clinical condition secreted exosomes are promising to develop into therapeutic products for wound healing treatment.

## Introduction

Wound healing is a complex process to restore the structure and function of damaged tissues. The healing process is divided into four overlapping phases of haemostasis, inflammation, proliferation, and remodeling, that are strictly regulated by multiple diverse growth factors, cytokines, enzymes and structural matrix proteins generated by multiple cell types such as dermal fibroblasts, epidermal keratinocytes, and immune cells (Sonnemann and Bement, 2011). Many treatment methods have been proposed for wound healing including cell therapy (Kanji and Das, 2017; Kosaric et al., 2019). Recently, mesenchymal stem cells (MSCs), which are multipotent stem cells derived from a variety of tissues, have emerged as a promising candidate to develop innovative therapeutic treatment for wound healing due to their ability to differentiate into the wounding cells, such as fibroblasts (Jahoda and Reynolds, 2001), or secrete many wound healing-promoting growth factors (Hu et al., 2018). Several factors secreted by MSCs with strong wound-healing potentials have been reported, including epidermal growth factor (EGF); interleukin-like growth factor (IGF); fibroblast growth factor (FGF); platelet-derived growth factor (PDGF); transforming growth factor (TGF); vascular endothelial growth factor (VEGF); interleukins (IL); interferon (IFN); stromal cell-derived factor-1 (SDF-1); and, tumor necrosis factor α (TNFα) (Hu et al., 2018). Interestingly, it is evidenced that the MSC secreted factors enveloped in enclosed vesicles named extracellular membrane vesicles (EVs) (Phinney and Pittenger, 2017).

Extracellular vesicles (EVs) are enclosed by a lipid-bilayer membrane and secreted by various cell types. The EVs are found in body fluids such as saliva, plasma, breast milk, amniotic fluids, and urine and in cell culture medium (Hao et al., 2006; Keller et al., 2007; Michael et al., 2010; Rupp et al., 2011; Zonneveld et al., 2014; Than et al., 2018). Importantly, these EVs, especially exosomes, enveloped with biological molecules that reflect physiological and pathological characteristics of the secreting cells during the biogenesis (Van Niel et al., 2003; Lee et al., 2011; Crescitelli et al., 2013; Bebelman et al., 2018; Bu et al., 2019). In wound healing, and MSC-derived EVs originated from human induced pluripotent stem cells and human bone marrow have been described to promote different biological events such as cell proliferation, cell migration and angiogenesis (Than et al., 2017). By inducing the expression of HGF, IGF1, NGF, and SDF1 which then activate Akt, Erk, and Stat3 signaling, MSC-derived EVs from ADMSC, UCMSC, and BMMSC tissues promoted cell migration, cell proliferation, collagen production, and the creation and maturation of newly formed vessels in addition to increased re-epithelialization and reduced scar widths (Shabbir et al., 2015; Zhang et al., 2015a; Hu et al., 2016; Vrijsen et al., 2016).

MSC EV's cargo are not static, but rather a product affected by many biophysical parameters. For example, EVs derived from various MSC tissue origins might have different quality and therapeutic effects due to the differences in parental MSC characteristics and potentials. Additionally, changes in MSC culture condition, cell seeding density, MSC passage or period of time that EV can be collected can also influence secretion profiles by MSCs, such as the yield of EVs and their contents. Thus, a wide range of conditions needs to be explored in attempts to increase MSC-EV yields and to control their contents.

To our knowledge, the differences in contents and the capacity to heal the wound of EVs isolated from three different human MSC sources of adipose tissue (AD), bone marrow (BM), and umbilical cord (UC) have not been evaluated. Thus, in this study, we investigated the level of some growth factors important to wound healing process carried by MSC-derived exosomes originated from BM, AD, and UC. Additionally, we evaluated the capacity of these primary human MSC-derived exosomes originated from the three tissues under serum- and xeno-free condition to stimulate epidermal keratinocyte and dermal fibroblast proliferation and migration. The data showed us the differences in growth factor enrichment and capacity of each exosome sources in wound healing. Understanding the MSC-derived exosomes associated with tissue origin will help to a better understanding of condition affecting exosome's contents and their roles in tissue regeneration.

## Materials and Methods

### Ethical Approval

Ethics approval for collection and use of human dermal fibroblasts and mesenchymal stem cells from adipose tissue, bone marrow, and umbilical cord was approved by the ethics committee of Vinmec International General Hospital Joint Stock Company. Donors provided their written informed consent to donate their samples in this study. The human tissue was collected from healthy donors with unknown gender and ages are from 20 to 60. All MSCs were culture in serum- and xeno-free MSC culture media.

### Mesenchymal Stem Cell Isolation and Culture

#### Adipose Tissue-Derived MSC Isolation and Culture

Adipose tissues (AD) were sectioned from abdominal area by surgical doctor at Vinmec International Hospital and transferred to the laboratory. The tissues were minced and digested by 200 U/mL collagenase type I solution (Gibco, Massachusetts, USA) and 0.1% human albumin solution for 1 h at 37°C with shaking. Cells were pelleted by a centrifugation at 500 × g for 10 min and supernatant was discarded. Subsequently, pellets were resuspended in the MSC culture media (StemMACS^TM^ MSC Expansion Media, Miltenyi Biotec, Bergisch Gladbach, Germany) and then centrifuged at 300 × g for 5 min to collect MSCs. The harvested MSCs then seeded into cell culture flask (Nunc, Thermo Scientific, Massachusetts, United States) coated with solution CTS^TM^ CELLstart^TM^ substrate (diluted in PBS at ratio 1: 300) (Gibco, Massachusetts, USA) with initial density of 3,000–5,000 cells/cm^2^ in the MSC culture media and cultured at 37°C and 5% CO_2_. The media were replaced by every 2 days of culture. When the cells reached 80% confluency, the cells were split by 0.05% trypsin for the next passage.

#### Bone Marrow-Derived MSC Isolation and Culture

Bone marrow (BM) was aspirated from the bilateral posterior iliac crest under general anesthesia. A total of 30 mL of BM was collected using a 10 mL syringe with heparin sodium as anticoagulants. The mononuclear cells-enriched fraction was separated using the density gradient centrifugation on Ficoll-Paque Premium (GE Healthcare Life Sciences, Pennsylvania, USA). After Ficoll separation, the mononuclear cells were plated into T75 cell culture flasks (Nunc, Thermo Scientific, Massachusetts, United States) coated with solution CTS^TM^ CELLstart^TM^ substrate (diluted in PBS at ratio 1: 300) (Gibco, Massachusetts, USA) with the density 22.5 × 10^6^ cells/flask. Cells were expanded in the MesenCult^TM^ ACF Plus Medium (StemCell Technologies, Vancouver, Canada) at 5% CO_2_ and 37°C. After 48 h, media containing non-adherence cells was replaced with new media for cell growth. When the cells reached 80% confluency, the cells were split by 0.05% trypsin for the next passage.

#### Umbilical Cord-Derived MSC Isolation and Culture

Umbilical cords (UC) were collected from pregnant women after baby delivery at the surgery of Vinmec International Hospital. The UCs were washed three times with ethanol 70% and then 3–5 times with PBS to sterilize and remove blood. The UC was cut into small pieces and transferred into a 50 mL conical tube following by an incubation with 500 U collagenase Type I solution (Gibco, Massachusetts, USA) for 1 h at 37°C with shaking. After digestion, solution was diluted in chilled PBS and centrifuge at 1,400 × g/10 min at 4°C to collect cell pellet. The cell pellet was resuspended in a MSC culture media (StemMACS^TM^ MSC Expansion Media, Miltenyi Biotec, Bergisch Gladbach, Germany) and transferred into cell culture flasks (Nunc, Thermo Scientific, Massachusetts, United States) coated with solution CTS^TM^ CELLstart^TM^ substrate (diluted in PBS at ratio 1: 300) (Gibco, Massachusetts, USA) and incubated at 37°C and 5% CO_2_ for cell expansion. With each 5 mg of UC, cell pellets were seed into 1 T25 cell culture flask. Culture media were replaced by every 2 days of culture. When the cells reached 80% confluency, the cells would be split by 0.05% trypsin for the next passage.

### Human Primary MSC Marker Analysis

Human primary MSCs isolated from AD, BM, and UC were examined for their MSC markers using Human MSC Analysis Kit (BD Biosciences, California, US) at the passage 3 when supernatant was harvested for exosome isolation. The kit includes the MSC positive cocktail (FITC CD90, PerCP-Cy™5.5 CD105, and APC CD73) and the negative MSC cocktail (PE CD45, PE CD34, PE CD11b, PE CD19, and PE HLA-DR). Flow cytometry was performed using a Beckman Coulter flow cytometer and Navious software.

### Cellular Senescence Analysis of Human Primary MSCs

Cells, including UCMSCs, ADMSCs, and BMMSCs, at passage three were analysis for cellular senescence using Senescence Cells Histochemical Staining Kit (Sigma-Aldrich, Missouri, USA). The procedures were performed following to the manufacturer's instructions. Briefly, cells were seeded in a six-well plate at 0.2 × 106 cells/well and incubated overnight at 37°C and 5% CO_2_. After incubation, culture medium was removed and cells were washed twice with 1X PBS. After that, cells were fixed with 1X Fixation Buffer for 6 min at room temperature prior to being rinsed three times with 1X PBS, and then incubated in Staining Mixture overnight at 37°C without CO_2_. The cells were washed twice with 1X PBS and stained with DAPI Staning Solution (Abcam, Cambridge, UK) for 5 min at room temperature. After staining, cells were washed twice with 1X PBS, examined under an inverted microscope IX73 (Olympus, Tokyo, Japan) and images were captured with a digital color camera. The images were semi-qualitatively analyzed with Image J software (version 1.46r).

### EV Production and Isolation

The ADMSCs, BMMSCs, and UCMSCs at passage three were maintained for EV generation. All three cell types were processed the same manner; but, ADMSCs and UCMSCs were cultured in StemMACS^TM^ MSC Expansion Media (Miltenyi Biotec, Bergisch Gladbach, Germany) and BMMSCs were cultured in MesenCult^TM^ ACF Plus Medium (StemCell Technologies, Vancouver, Canada). The cells at the passage two were split and seeded into the new flasks, which had been coated with solution CTS^TM^ CELLstart^TM^ substrate (diluted in PBS at ratio 1: 300) (Gibco, Massachusetts, USA), for passage 3 to generate EVs. The cells then were incubated at 37°C and 5% CO_2_ for ~4–5 days to reach 80% confluency. During the incubation, cell culture media were not renewed for the cells to secrete EVs into the supernatant. Supernatant, which contained EVs, was collected when the cells reach 80% confluency and centrifuged at 300 × *g* for 10 min at 4°C to remove cell debris, then at 2,000 × *g* for 10 min to remove apoptotic bodies, and followed by at 10,000 × *g* for 30 min at 4°C to remove microvesicles. Exosomes (EXs) were collected by a centrifuge at 100,000 × *g* for 70 min at 4°C (Optima XPN-100 Ultracentrifuge, Beckman Coulter, California, USA). The EX pellets were resuspended and washed in PBS and concentrated again at 100,000 × g/70 min at 4°C for cleaned EX harvest. The cleaned EXs were resuspended in 100 μL PBS and stored at −80°C for further uses.

### Protein Extraction

A volume of EXs was mixed with an equal volume of RIPA extraction buffer in Protein Lo-Bind tubes (Eppendorf, Hamburg, Germany) and shaken for 15 min at room temperature. The resulting mixtures were centrifuged at 14,000 × *g* for 15 min at 4°C, and the protein supernatant decanted and stored at −20°C until required.

### Western Blot

Total exosome protein (10 μg/lane) were separated by 4–12% SDS-PAGE gels (Invitrogen, USA) at 200 V for 35 min at 4°C. Proteins were then transferred to PVDF membrane (AmershamTM, GE Healthcare Life Sciences, Illinois, US) at 200 mA for 2 h at 4°C prior to being blocked with 5% skimmed milk in TBST buffer for 1 h. The membrane was probed with diluted primary antibodies against CD9, CD63 (Santa Cruz Biotechnology, Texas, US), AGO2 (Abcam, Cambridge, UK) and Tubulin (Thermo Scientific, Massachusetts, US) overnight at 4°C and then incubated with secondary antibodies (Amersham ECL Mouse IgG, HRP-linked whole Ab, GE Healthcare Life Sciences, Pittsburgh, USA). Antibody binding was detected with ECL chemiluminescence substrate (Sigma-Aldrich, Singapore) and imaged on ImageQuant LAS 500 (GE Healthcare Life Sciences, Illinois, US).

### Transmission Electron Microscopy (TEM)

Exosome samples were fixed with 4% paraformaldehyde and then deposited onto Formvar-carbon coated grids (Ted Pella Inc., California, USA). Samples were washed eight times with PBS prior to being stained with uranyl-oxalate. The grids were let dried at room temperature. Imaging was performed using a JEOL 1,100 Transmission Electron Microscope (TEM, JEOL Ltd., Tokyo, Japan) at 80 kV.

### Growth Factor Analysis Using Luminex Assay

Growth factors such as fibroblast growth factor 2 (FGF-2), hepatocyte growth factor (HGF), platelet-derived growth factor-BB (PDGF-BB), vascular endothelial growth factor A (VEGF-A), and transforming growth factor beta (TGF-β) were measured by Luminex assay using ProcartaPlex^TM^ Multiplex Immunoassays (Human Custom ProcartaPlex 4—Plex Kit and ProcartaPlex Human TGF beta 1 Simplex Kit—Custom, ThermoFisher, Massachusetts, US). Frozen exosome suspension was thawed and kept on ice for sample preparation following the manufacturer's instruction. The luminescent signal was detected using LuminexTM 100/200^TM^ system with xPONENT 3.1 software.

### Proliferation Assay

Human dermal fibroblasts and keratinocytes (HaCaT) were seeded into a 96-well plate (5,000 cells/well) with culture medium (5% FBS and 1% Pen/Strep in DMEM/F12) containing exosomes with three different doses of 1, 10, and 20 μg total exosomal proteins/1 mL depleted media. Depleted medium was used as control group which fetal bovine serum (FBS) was centrifuged at 100,000 × g for 27 h to removed FBS vesicles.

Cells were incubated at 37°C and 5% CO_2_ overnight for attachment before proliferation analysis using 3-(4,5-dimethylthiazol-2-yl)-2,5-diphenyl tetrazolium bromide (MTT) assay kit (Abcam, Cambridge, UK). Step by step was performed followed instruction by manufacture. The proliferation of cell was measured at time points of 0 h (as control) and 48 h.

### Cell Migration Assay

Human dermal fibroblasts and keratinocytes (HaCaT) were seeded into a 24-well plate with culture medium (5% FBS and 1% Pen/Strep in DMEM/F12) with the density of 2 × 10^5^ fibroblasts/well and 3 × 10^5^ keratinocytes/well to obtain 100% confluency. Cells were incubated at 37°C and 5% CO_2_ to attach to the bottom of cell culture plate. Then, the cells were incubated with Mitomycin C (10 μg/mL) for 2 h to inhibit cell proliferation. The cells were washed with DMEM twice prior to creating a scratch. Detached cells were removed by washing the wells with DMEM for twice. The cells were added fibroblast culture medium containing exosomes with three different doses of 1, 10, and 20 μg total exosomal protein/mL. Depleted medium was used for the control group which FBS was centrifuged at 100,000 × g/27 h to removed FBS vesicles. Cell migration was observed and captured by inverse microscope (Canon, Tokyo, Japan) with 4X magnification for different time points. Rate of cell migration to close wounded area was analyzed using ImageJ software (version 1.48).

### Statistical Analysis

All statistical analyses were performed using R software version 3.4.4. The statistically significant differences between groups and were assessed by *T*-Test and Two-Way ANOVA and Tukey HSD tests. *p* < 0.05 was considered as statistical significance. All data were shown as means ± SD.

## Results

### MSC Cell Culture and Cellular Senescence

**Figure 1:**
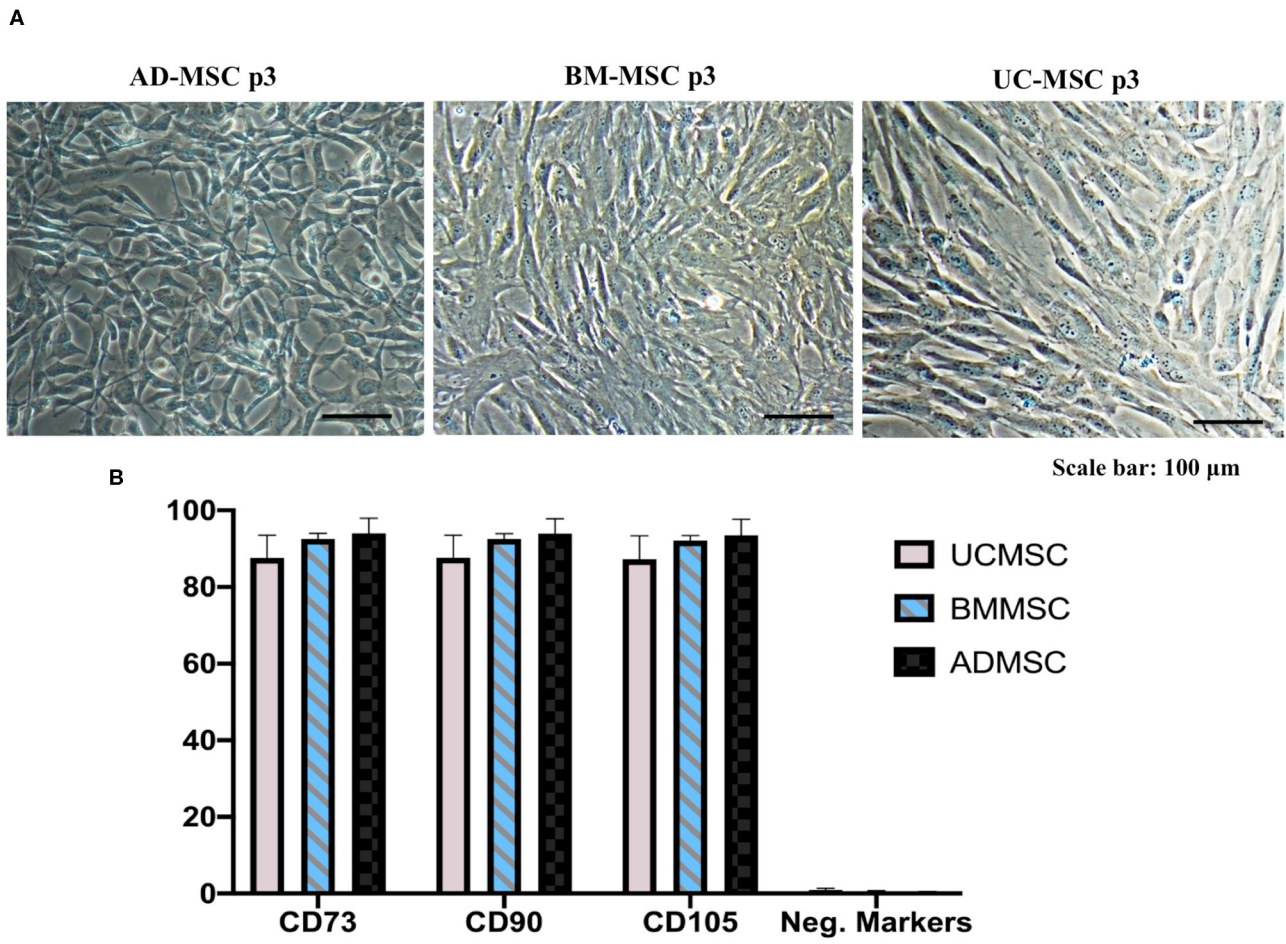
Human primary mesenchymal stem cells isolated from AD, BM, and UC at passage 3 of cell culture. **(A)** Typical morphology of MSCs was captured under Nikon Inverted Microscope Eclipse Ti-S. **(B)** Expression of MSC markers (*n* = 5) were analyzed using flow cytometry approach and Human MSC Analysis Kit (BD Biosciences). Positive markers include CD90, CD105, and CD73, and negative markers include CD45, CD34, CD11b, CD19, and HLA-DR. ADMSC, Adipose tissue-derived MSCs; BMMSC, Bone marrow-derived MSCs; UCMSC, Umbilical cord-derived MSCs. Error bars indicate ± SD.

We evaluated the morphology and cell surface markers of passage three human primary MSCs originated from AD, BM, and UC at the time point of supernatant collection by microscopy and Flow Cytometry. We observed similar morphology in all three evaluated ADMSCs, BMMSCs, and UCMCSs (Figure 1A). Additionally, all MSCs originated from AD, BM, and UC expressed highly MSC positive markers of CD90, CD105, and CD73 (~90%) and very low MSC negative markers of CD45, CD34, CD11b, CD19, and HLA-DR (<1%) (Figure 1B).

**Figure 2:**
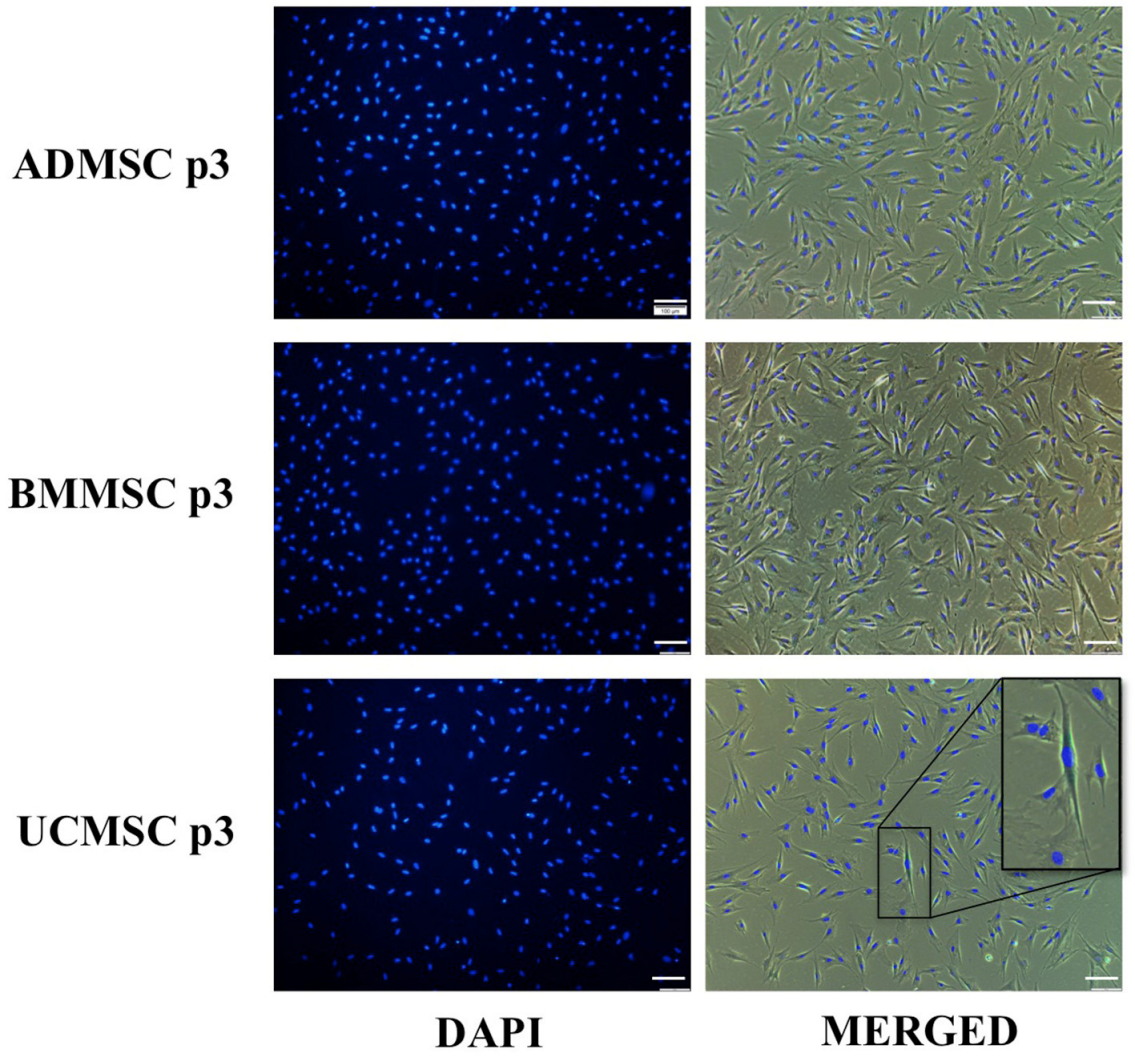
Cellular senescence characteristics of human primary ADMSCs, BMMSCs, and UCMSCs at the passage three under serum- and xeno-free culture condition. Three cell types were analyzed for the cellular senescence using Senescence Cells Histochemical Staining Kit (Sigma-Aldrich). Results indicated that only UCMSCs expressed 0.18% cells with senescence signals and BMMSCs and UCMSCs did not expressed any cellular senescence signal.

Additionally, we evaluate the cellular senescence of three ADMSCs, BMMSCs, and UCMCSs at the passage three to understand the cell state at the time of secreting exosomes. Data showed that only UCMSCs expressed their cellular senescence with 0.18%, but there was no senescence signs observed in ADMSCs and BMMSCs (Table 1; Figure 2).

**Table 1:** Percentage of MSCs at the passage three exhibited the cellular senescence.

**Cell type**	**Mean of cells expressed senescence (%)**
ADMSCs	0
BMMSCs	0
UCMSCs	0.180349236 ± 0.075

### Characterization of Exosomes Originated From ADMSCs, BMMSCs, and UCMSCs

**Figure 3:**
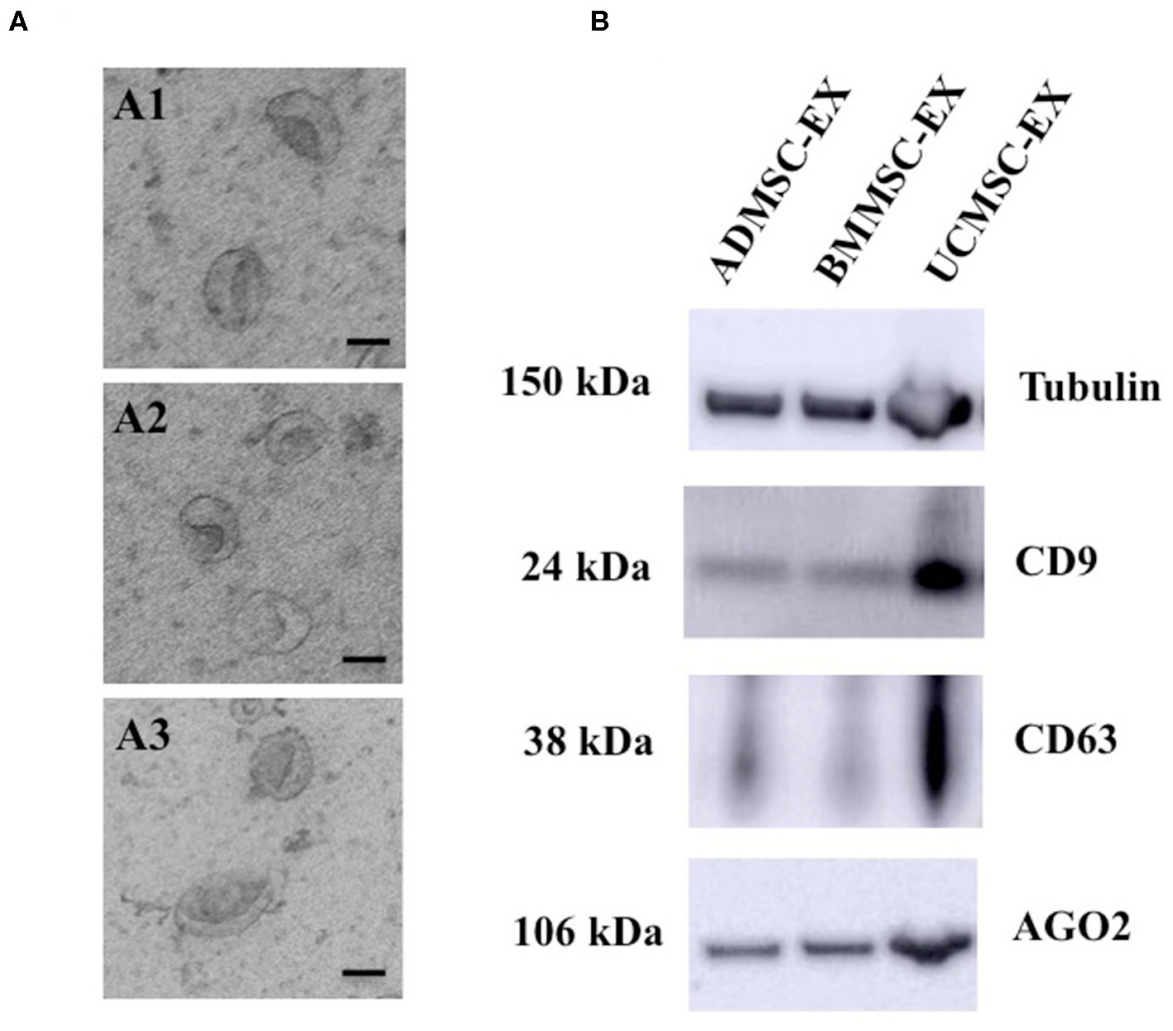
Morphology and marker analysis of exosomes derived from human primary ADMSCs, BMMSCs, and UCMSCs. **(A)** Morphology analysis of exosomes observed under TEM showed all exosomes have a cup-shape morphology (Scale bar: 100 nm): (A1) A representative of ADMSC-derived exosomes, (A2) A representative of BMMSC-derived exosomes, (A3) A representative of UCMSC-derived exosomes. **(B)** Exosomal proteins, 10 μg total protein were loaded each lane, including CD9, CD63, AGO2 and Tubulin, enriched in exosomes released from ADMSCs, BMMSCs, and UCMSCs. ADMSC-EX, ADMSC-derived exosomes; UCMSC-EX, UCMSC-derived exosomes; BMMSC-EX, BMMSC-derived exosomes. Data are representative of at least three independent experiments. Scale bars indicate 100 nm.

We analyzed the morphology of exosomes generated from three MSC sources using transmission electron microscope (TEM). The vesicles showed homogeneous cup-shape morphology and a size range ~200 nm (Figure 3A). Additionally, we analyzed proteins are expected to enrich in exosomes such as transmembrane proteins (CD9 and CD63) and intracellular protein (AGO2). Results showed that exosomes from three ADMSCs, BMMSCs, and UCMSCs expressed CD9, CD63, AGO2 and Tubulin (internal control) (Figure 3B). These indicated that exosomes were secreted by MSCs originated from AD, BM, and UC under xeno- and serum-free culture condition.

### Growth Factor Expression in Different Human Primary MSC-Derived Exosomes

In order to compare the secretome characteristic of human primary MSC-derived exosomes originated from AD, BM, and UC tissues, we evaluated a panel of growth factors including VEGF-A, FGF-2, HGF, PDGF-BB, and TGF-β1 using Luminex assay (Table 2). Protein levels enriched in exosomes were reported as mean of biological replicates, which were calculated and equivalent to 1 × 10^6^ secreting cells. Results showed that BMMSC-derived exosomes highly expressed VEGF-A, FGF-2, and PDGF-BB (>10 pg/10^6^ cells, *p* < 0.01) in comparison to exosomes generated from UCMSCs and ADMSCs. However, VEGF-A was detected with the greatest level in exosomes released from ADMSCs (12.65 pg/10^6^ cells, *p* < 0.05), while other growth factors were detected with lower levels (<8 pg/10^6^ cells). All growth factors enriched similarly at low level in UCMSC-derived exosomes.

**Table 2:** Quantity of growth factors in exosomes released from human primary MSC at the passage three.

**Growth factors**	**UCMSC-derived EX (*n* = 5)**	**BMMSC-derived EX (*n* = 6)**	**ADMSC-derived EX (*n* = 4)**
VEGF-A (pg)	4.79 ± 5.63	12.15 ± 5.28	12.65 ± 8.88
FGF-2 (pg)^*^	1.02 ± 0.7^*^	13.66 ± 7.71^*^	4.72 ± 1.15
HGF (pg)^***^	1.47 ± 0.49^***^	2.84 ± 1.6^**^	7.19 ± 1.01^**^
PDGF-BB (pg)	1.64 ± 0.39	10.43 ± 11.55	1.56 ± 1.30
TGF-β (pg)^****^	3.61 ± 0.69	0	0

Statistical significance was determined by ANOVA and post-hoc Tukey HSD tests, and is indicated by:

* where p < 0.05;

** where p < 0.01;

*** where p < 0.001;

**** *where p < 0.0001. ± indicate ± SD (Standard Deviation)*.

Interestingly, we detected TGF-β only in exosomes released by UCMSCs, but not by in exosomes released by BMMSCs or ADMSCs. Other growth factors, including VEGF-A, FGF-2, HGF, and PDGF-BB, were detected in exosomes secreted by all three ADMSCs, BMMSCs, and UCMSCs. Among these four growth factors, VEGF-A and PDGF-BB did not expressed differently between three exosome sources while FGF-2 and HGF was different. For example, BMMSC-derived exosomes expressed greatest amount of FGF-2 (13.66 pg/10^6^ cells) and UCMSC-derived exosomes expressed the lowest amount of the protein (1.02 pg/10^6^ cells) (*p* < 0.05). Moreover, HGF was detected with the highest amount in ADMSC-derived exosomes while the lowest amount in UCMSC-derived exosomes (*p* < 0.001). These data indicated that amount of exosomal growth factors depends on the secreting cell origin.

### Capacity of Human Primary MSC-Derived Exosomes in Inducing Cell Proliferation

**Figure 4:**
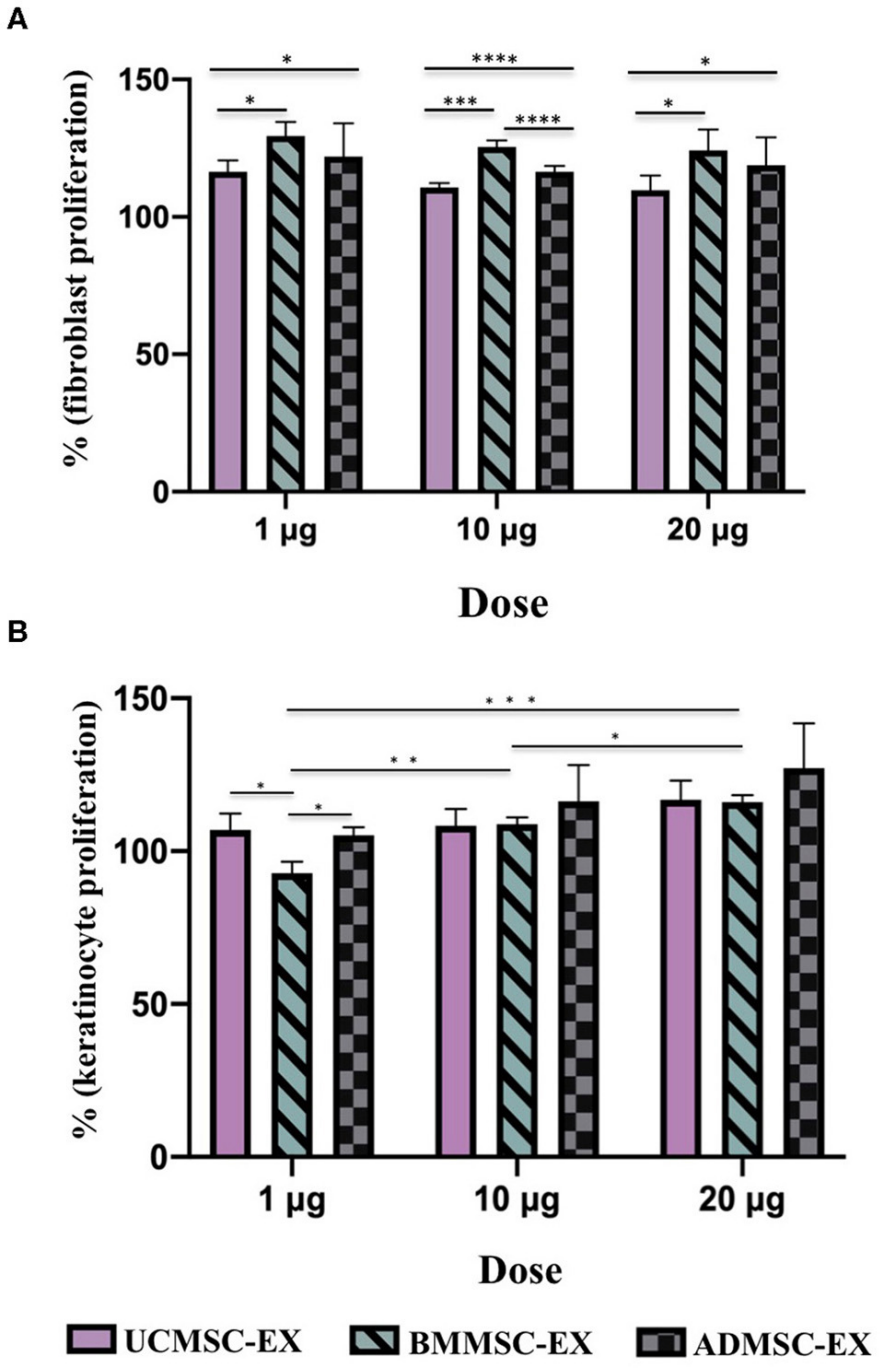
Capacity of primary dermal fibroblast and keratinocyte proliferation under stimulation of exosomes released by BMMSCs, ADMSCs, and UCMSCs cultured under serum- and xeno-free condition. Percentage of cell proliferation was normalized to the control (100%). **(A)** Fibroblast proliferation under exosome stimulation: There were differences between exosomes released from three sources of BMMSCs, ADMSCs, and UCMSCs within the same dose, in which BMMSC-derived exosomes have the greatest enhancement in the primary dermal fibroblasts. There was no difference in cell proliferation stimulation associated with doses, especially different doses of BMMSC-derived exosomes and ADMSC-derived exosomes, excepted for UCMSC-derived exosomes that dose of 20 μg expressed lower stimulation on cell proliferation compared to dose of 1 μg. **(B)** Keratinocyte proliferation under exosome stimulation: A stronger induction of keratinocyte proliferation was observed in the cell treated with the higher dose of exosomes, especially BMMSC-derived exosomes. With the dose of 1 μg exosomal protein, ADMSC- and UCMSC-derived exosomes exhibited a stronger capacity to enhance keratinocyte proliferation in comparison with UCMSC-derived exosomes which exhibited the lowest capacity to stimulate the proliferation. BMMSC-EX, BMMSC-derived exosomes; ADMSC-EX, ADMSC-derived exosomes; UCMSC-EX, UCMSC-derived exosomes. Statistical significance was determined by ANOVA and *post-hoc* Tukey HSD tests, and is indicated by: * where *p* < 0.05; ** where *p* < 0.01; *** where *p* < 0.001; **** where *p* < 0.0001.

In order to investigate the capacity of human primary MSC-derived exosomes to induce cell proliferation, we performed colorimetric MTT assay to evaluate the effect of MSC-derived exosomes from AD, BM, or UC sources in dermal fibroblast and keratinocyte proliferation. In general, all MSC-derived exosomes originated from the three sources enhanced fibroblast proliferation. However, the cell proliferation enhancement of exosomes was dependent on cell sources and exosome doses (Figure 4). Regarding fibroblast proliferation, MSC-derived exosomes originated from BM showed the greatest influence on dermal fibroblast proliferation at any dose of exosomes (*p* < 0.05), while UCMSC-derived exosomes exhibited the lowest enhancement to cell proliferation (*p* < 0.05) (Figure 4A). Interestingly, the higher dose of exosomal proteins (20 μg) the lower proliferation enhancement was observed in UCMSC-derived exosome (1 μg) (*p* < 0.05). On the other hand, the ability of BMMSC- and ADMSC-derived exosomes to induce cell proliferation is dose-independent (Figure 4A). These data indicated that exosomes secreted from all three BMMCSs, ADMSCs, and UCMSCs have capacity to induce primary dermal fibroblast proliferation, but BMMSC-derived exosomes exhibited superior effect among the three.

Additionally, keratinocyte proliferation have influenced by exosome dose (Figure 4B). The higher dose of exosomes, the stronger induction of keratinocyte proliferation was observed, especially BMMSC-derived exosomes (*p* < 0.001). In regard to other exosomes, the proliferation was higher in the keratinocytes treated with 10 μg ADMSC-derived exosomes compared to the keratinocytes treated with 1 μg ADMSC-derived exosomes (*p* < 0.05), and keratinocytes treated with 20 μg UCMSC-derived exosomes exhibited a greater proliferation rate compared to the keratinocytes treated with dose of 1 μg UCMSC-derived exosomes (*p* < 0.01). However, the association of keratinocyte proliferation with exosome origins was not clear as the difference was only observed in the low exosomal dose of 1 μg (Figure 4B). With the dose of 1 μg exosomal protein, ADMSC- and UCMSC-derived exosomes exhibited a stronger capacity to enhance keratinocyte proliferation in comparison with UCMSC-derived exosomes (*p* < 0.05). Thus, these data indicated that keratinocyte proliferation is influenced by both exosome dose and origin.

### Capacity of Human Primary MSC-Derived Exosomes in Cell Migration

**Figure 5:**
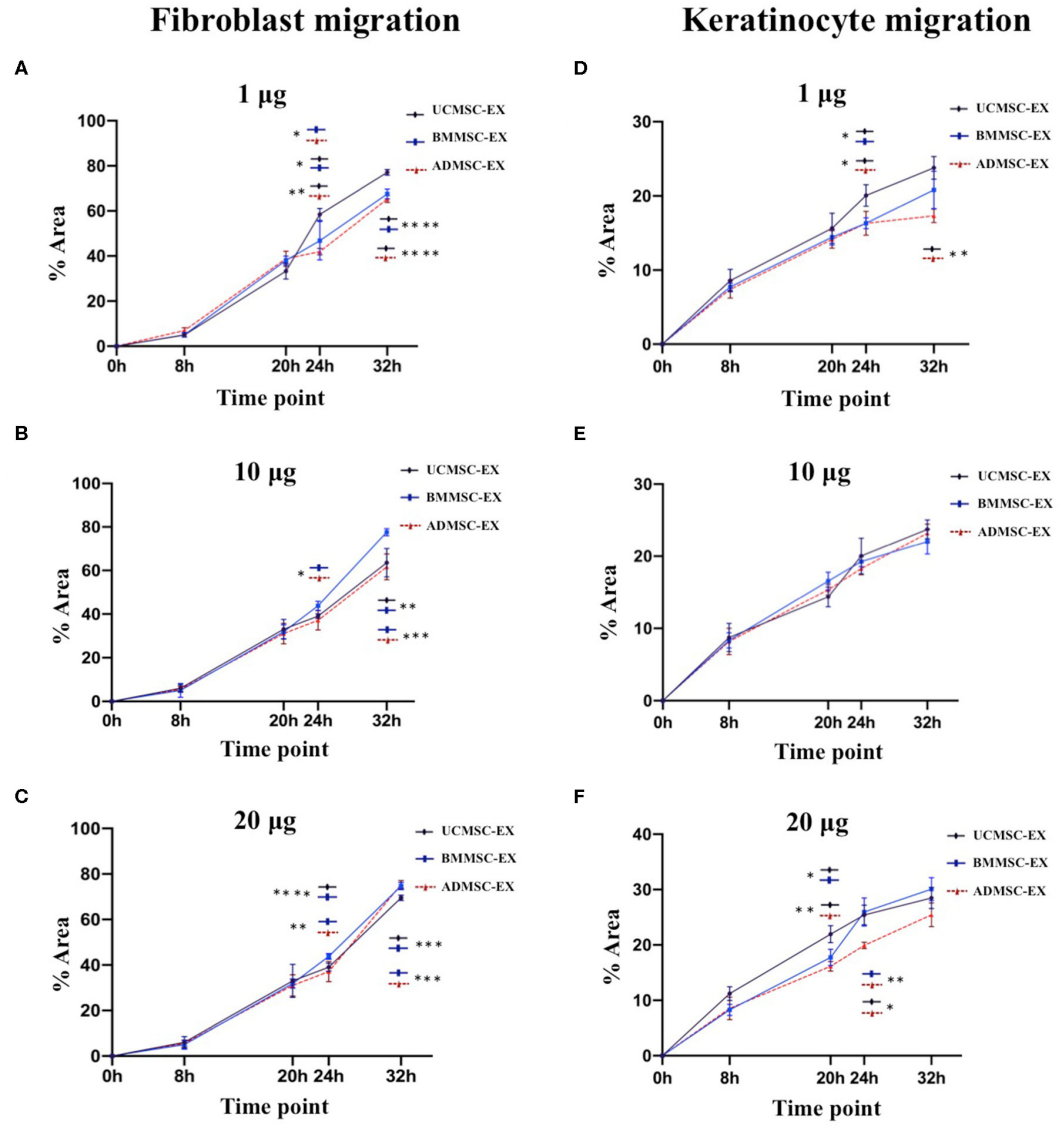
Primary dermal fibroblast and keratinocyte migration under stimulation of exosomes released from BMMSCs, ADMSCs, and UCMSCs. Exosomes released by three cell sources were added to scratched primary dermal fibroblast and keratinocyte cultures to final total exosomal protein concentration of 1 μg **(A,D)**, 10 μg **(B,E)** or 20 μg **(C,F)** / 0.1 mL depleted culture media. The primary fibroblasts and keratinocytes were incubated at 5% CO_2_ and 37°C and allowed to migrate with images captured at different time points. Image analysis was performed using ImageJ and data are presented as mean percent area of wound coverage in μm^2^ ± SD, from at least 3 independent biological replicates. Data showed that BMMSC-derived exosomes exhibited a greater induction on fibroblast migration within the doses of 10 and 20 μg **(B,C)**, whereas UCMSC-derived exosomes exhibited the greater induction on fibroblast migration within the dose of 20 μg exosomal protein **(A)**. Regarding to keratinocyte migration, UCMSC-derived exosomes showed a strongest stimulation on cell migration **(D,F)**, in addition to the higher doses of exosomal protein derived from all three MCSs exhibited the higher stimulation on keratinocyte migration. Statistical significance was determined by ANOVA and *post-hoc* Tukey HSD tests, and is indicated by: * where *p* < 0.05; ** where *p* < 0.01; *** where *p* < 0.001; **** where *p* < 0.0001. BMMSC-EX, BMMSC-derived exosomes; ADMSC-EX, ADMSC-derived exosomes; UCMSC-EX, UCMSC-derived exosomes.

**Figure 6:**
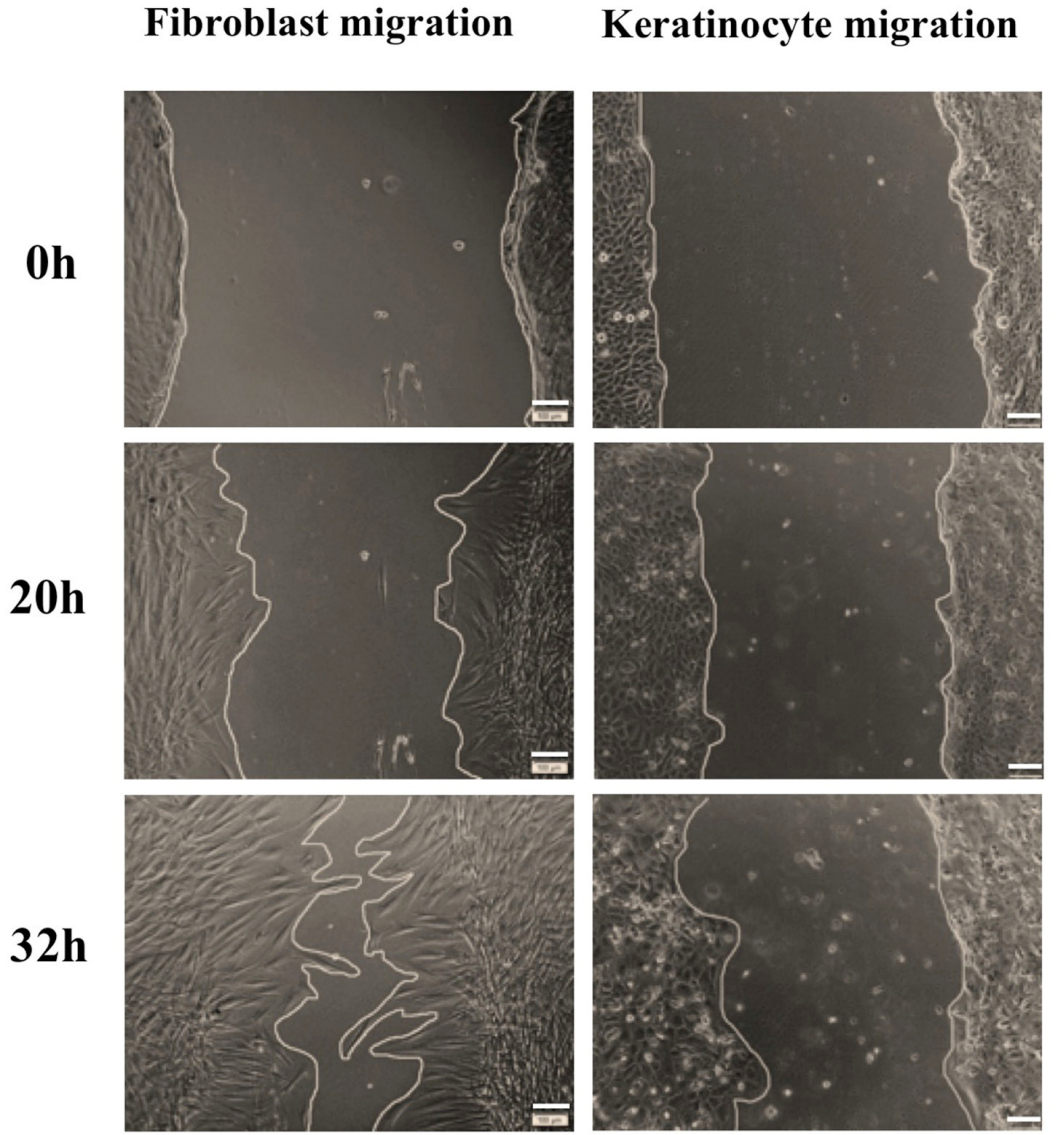
Representative images of fibroblast and keratinocyte migration under stimulation of BMMSC-derived exosomes within the dose of 20 μg exosomal protein. The primary fibroblasts and keratinocytes were seeded with 2 × 10^5^ and 3 × 10^5^ cells/well, respectively, and inhibited cell proliferation using mitomycin C. A wound scratch was created for each well and cells were imaged at different time points. In comparison to keratinocytes, fibroblasts migrated faster to close the wound. Scale bars indicate 100 μm.

In order to examine the ability of MSC-derived exosomes to regulate cell migration, we cultured human primary dermal fibroblasts and keratinocytes and investigated for cell migration using wound scratch assay. Exosomes from three cell sources showed similarity in their ability to stimulate cell migration in dose dependent manner until 20 h for fibroblasts and 32 h for keratinocytes (Figures 5, 6). After that, the induction of fibroblast migration was not similar between three cell-derived exosomes. The UCMSC-derived exosomes at a dose of 1 μg exhibited a greater influence on cell migration after 24 h (*p* < 0.05 and *p* < 0.01) and 32 h (*p* < 0.0001) compared to BMMSC- and ADMSC-derived exosome (Figure 5A). However, BMMSC- and ADMSC-derived exosomes expressed higher capacity to enhance cell migration within doses of 10 and 20 μg (*p* < 0.0001, 32 h, BMMSC-derived exosomes; *p* < 0.01, 32 h, ADMSC-derived exosomes) (Figures 5B,C). It is noted that BMMSC-derived exosomes showed the greatest stimulating capacity on cell migration within the dose of 10 μg (*p* < 0.01 and *p* < 0.001) and 20 μg (*p* < 0.001) at 32 h (Figures 5B,C). These indicated that the capacity of exosomes to induce dermal fibroblast migration might be affected by doses and secreting cell origin.

The dose dependent manner of cell migration was observed clearly in keratinocytes treated with all exosomes derived from three tissues. In this niche, keratinocytes treated with 20 μg exosomal protein exhibited the greatest migration rate and keratinocytes treated with 1 μg exosomal protein exhibited the lowest migration rate in all three exosomes derived from ADMSCs (*p* < 0.01), BMMSCs (*p* < 0.01), and UCMSCs (*p* < 0.05) at 32 h. When examination the association of keratinocyte migration with exosome origins, data indicated that UCMSC-derived exosomes have a greater capacity in stimulating the cell migration. For example, UCMSC-derived exosomes stimulated keratinocytes to migrate faster at 24 h (*p* < 0.05) and 32 h (*p* < 0.01) at within the dose of 1 μg (Figure 5D) and at 20 h (*p* < 0.05 and 0.01) and 24 h (*p* < 0.05 and 0.01) within dose of 20 μg (Figure 5F). These indicated the influence of exosomes on keratinocyte migration related to both exosome origin and the exosomal protein dose that UCMSC-derived exosomes and the high dose of 20 μg exosomes have a greatest stimulation.

## Discussion

Growing evidence indicates the importance of mesenchymal stem cell-derived exosomes in therapeutic purposes. Exosomes from ADMSCs, BMMSCs, and UCMSCs have been showed to be involved in many biological processes in different cell types due to its various components (Zhang et al., 2016; Rajendran and Gangadaran, 2017; Xu and Wang, 2017; Cho et al., 2018; Hong et al., 2019; Thomi and Surbek, 2019). For examples, human ADMSC-derived exosomes have been demonstrated to ameliorate atopic dermatitis (Cho et al., 2018), or modulate various cellular processes such as proliferation, migration, inflammatory response, and angiogenesis (Hong et al., 2019). Additionally, BMMSC-derived exosomes are able to enhance osteoclastogenesis (Xu and Wang, 2017), alleviate liver fibrosis (Rong et al., 2019), and promote angiogenesis (Ding et al., 2019). Furthermore, exosomes released by UCMSCs protect cells from oxidative stress-induced cell apoptosis *in vitro* (Zhang et al., 2016), and promote cutaneous wound healing (Zhang et al., 2015a) and human skin rejuvenation (Kim et al., 2017). Therefore, MSC-derived exosomes are promising candidate to develop effective therapeutic products. However, translating these promising results into clinical application requires a number of criteria, for instance safety. General cell culture medium includes animal components, such as growth factors and other nutrients, from FBS that support for normal cell development. However, these animal originated components are not allowed to apply for human. Therefore, this study evaluated the roles of exosomes released by primary human ADMSCs, BMMSCs, and UCMSCs under serum- and xeno-free culture medium in supporting fibroblast proliferation and migration in order to further developing clinical application in cutaneous wound healing treatment. We demonstrated that all three ADMSCs, BMMSCs, and UCMSCs under serum- and xeno-free culture condition secreted exosomes into conditioned media. The cell state of all three ADMSCs, BMMSCs, and UCMSCs are similar without signals of aging cells, except that only very low proportion of UCMSCs exhibited cellular senescence (0.18%). These exosomes expressed typical morphology of cup-shape with a size between 40 and 250 nm. Additionally, ADMSC-, BMMSC-, and UCMSC-derived exosomes expressed transmembrane and intracellular proteins, including CD9, CD63, and AGO2, which are expected to enrich in exosomes and considered as exosomal markers (Figure 3B). This indicated that under clinical culture medium, primary human MSCs secret exosomes which is consistent to normal culture conditions (Shabbir et al., 2015; Zhang et al., 2015a).

Previously, different molecules in exosomes were reported to affect target cell function (Park et al., 2019). Focusing on skin wound healing and regeneration, we evaluated several crucial growth factors of dermal skin layer in exosomes. In accordance with previous reports (Than et al., 2017; Hong et al., 2019; Zhu et al., 2019), we observed differential expression of growth factors associated with parental MSC sources originated from AD, BM, or UC tissues that might associated with further modulation of the target cells and tissues, including wound healing. For instance, exosomes bearing Jagged 1 released by fetal dermal MSCs activate Notch pathway that promotes fibroblast migration to close wounds (Wang et al., 2019). Or exosomes released by BMMSCs carrying microRNA-126 mediate PTEN to activate PI3K/AKT signaling pathway leading to stimulate angiogenesis (Ding et al., 2019). The loss of PTEN in fibroblasts causes skin fibrosis (Parapuram et al., 2011), thus the capacity of exosomes to modulate gene expression in skin cells is essential to drive a normal healing process. We showed that the crucial growth factors of skin wound healing-mediated biological processes (Desmoulière et al., 1993; Li et al., 2004; Nunes et al., 2016; Hu et al., 2019; Yin et al., 2019) including VEGF-A, FGF-2, HGF, PDGF-BB, and TGF-β (Table 2), were packaged into exosomes originated from BMMSCs, ADMSCs, and UCMSCs. These growth factors have been detected in exosomes from different cell types such as tumor cells and liver-derived MSCs (Nishida et al., 2006; Fouraschen et al., 2012; Katoh, 2013), and they are among main factors inducing vascular formation and contributing into other biological processes of skin (Park et al., 2019). Thus, exosomes produced according to this current study and their packaged factors may play important roles in wound healing process. Despite exosomes from MSCs have been demonstrated to play important roles in physiological state of cells and tissue, there have been only several investigations into growth factors packaged in ADMSC-, BMMSC-, and UCMSC-derived exosomes, even exosomes secreted in normal cell culture condition with FBS (Ahn et al., 2018; Han et al., 2019; Takeuchi et al., 2019). Therefore, it needs more studies in the field of molecules such as growth factors packaged in exosomes secreted by MSCs derived from BM, AD, and UC, especially in cell culture condition for future clinical application.

Fibroblasts and keratinocytes are key cells of dermal and epidermal layers and play important roles in skin, which fibroblasts play a major function in producing extracellular matrix components and important proteins for skin structure (Frazier et al., 1996; El Ghalbzouri and Ponec, 2004; Bainbridge, 2013) and keratinocytes are responsible for physical roles to prevent our body from toxin and pathogen invasion, heat and moisture loss, secreting cytokines and stimulating inflammation (Haake et al., 2001; Spiekstra et al., 2007). Previous studies showed that exosomes derived from human BMMSCs, ADMSCs, and UCMSCs cultured in FBS supplementing medium can induce keratinocyte and fibroblast proliferation, migration and angiogenesis (Shabbir et al., 2015; Zhang et al., 2015a,b; Hu et al., 2016, 2019; Than et al., 2017). In our study, all three MSC sources of BM, AD and UC cultured in serum- and xeno-free medium produced exosomes with the capacity to induce immortalized keratinocyte and human primary dermal fibroblast proliferation and migration (Figures 4, 5). However, the influence of exosomes derived from three tissues seems different and depends on the target cell type, for instances, ADMSC-derived exosomes have the strongest induction and UCMSC-derived exosomes have the lowest induction on fibroblast proliferation; but ADMSC- and UCMSC-derived exosomes have a similar induction level and higher than the induction of BMMSC-derived exosomes on keratinocyte proliferation (Figure 4). This situation was also observed in the cell migration for both cell types, which BMMSC-derived exosomes have a greater induction on fibroblast migration compared to UCMSC-derived exosomes have a greater induction on keratinocyte migration. Moreover, the exosome doses also affected the migration of both keratinocytes and fibroblasts; in addition to fibroblast migration is faster compared to keratinocyte migration. Therefore, capacity of exosomes to enhance cell proliferation and migration depends on all exosome origin, exosome doses and target cells of exosomes.

We observed a correlation between the growth factor levels of exosomes and their function in promoting cell proliferation and migration. For example, the TGF-β was only detected in UCMSC-derived exosomes which have the strongest stimulation on keratinocyte migration (dose 20 μg > dose 10 μg > dose 1 μg) and the lowest stimulation of fibroblast proliferation (Table 2; Figures 4, 5). Additionally, we detected the highest levels of FGF-2, HGF, and PDGF-BB growth factors in BMMSC-derived exosomes that exhibited highest capacity to induce migration (dose 20 μg > dose 10 μg) (Table 2; Figure 5). FGF members have showed to promote not only fibroblast proliferation but also migration and differentiation through binding to and activating fibroblast growth factor receptors, thereby having its application in tissue regeneration (Yun et al., 2010). Besides, HGF and PDGF-BB are important multifunctional growth factors for cell proliferation and mobility (Taniguchi et al., 2004; De Donatis et al., 2008; Sun et al., 2012; Gonzalez et al., 2017; Dai et al., 2018). However, TGF-β that is reported to regulate fibroblast proliferation through miR-21 modulating PTEN/AKT signaling pathway (Liu et al., 2016) or to induce ERK/MAPK signaling pathway to enhance migration of endometrial stromal cells (Liu et al., 2019) was not detected in BMMSC- and ADMSC-derived exosomes in this study. In this study, UCMSC-derived exosomes carry the highest level of TGF-β but have lowest level of fibroblast proliferation (Table 2; Figure 4). Thus, the point of TGF-β mechanism to regulate cell proliferation should be further investigated. Additionally, VEGF-A, which expressed highly in BMMSC- and ADMSC-derived exosomes and was not examined for its function in this current study, plays a key role in angiogenesis and its expression in endothelial cell were enhanced by exosomes released by human endothelial progenitor cells (Li et al., 2016). Thus, the MSC-derived exosomes may modulate cell proliferation, cell migration, and other biological processes associated with wound healing through different mechanisms. However, we did not investigated mechanism of exosomes used to regulate these processes in this current study. Future work will be required to characterize the exact role of growth factors enveloped in MSC-derived exosomes in activation of biological processes of cutaneous wound healing.

## Conclusions

In conclusion, this is the first study compared the growth factor content and wound healing capacity of exosomes released by primary human MSCs originated from three sources of bone marrow, adipose tissue and umbilical cord. Under serum- and xeno-free culture condition, all ADMSCs, BMMSCs, and UCMSCs secreted exosomes bearing VEGF-A, FGF-2, HGF, and PDGF-BB, but only UCMSC-derived exosomes carrying TGF-β. Exosomes secreted by three primary MSC sources promoted keratinocyte and dermal fibroblast proliferation and migration, with a superior capacity belongs to UCMSC-derived exosomes to keratinocytes and BMMSC-derived exosomes to fibroblasts. Moreover, the induction of cell proliferation and migration is a dose-dependent manner in addition to depend on exosome origin, target cells of exosomes and particular cellular processes. However, further studies are needed to investigate the mechanism of each MSC-derived exosomes in wound healing processes, especially regarding to the association of exosome origins and doses with target cells of exosomes.

## Data Availability Statement

The datasets generated for this study are available on request to the corresponding author.

## Ethics Statement

The studies involving human participants were reviewed and approved by The Ethics Committee of Vinmec International General Hospital Joint Stock Company. The patients/participants provided their written informed consent to participate in this study.

## Author Contributions

UT, X-HN, NH, and LT contributed to conceptualization. TN, DH, X-HN, H-PN, and UT contributed in the draft of project. TN, DH, MT, PTXD, VD, PTMD, H-PN, DV, and HB contributed in doing experiments and collecting data. UT, TN, DH, H-PN, X-HN, LT, NH, VD, PTMD, and HB contributed in interpreting data. All authors contributed to the article and approved the submitted version.

## Conflict of Interest

The authors declare that the research was conducted in the absence of any commercial or financial relationships that could be construed as a potential conflict of interest.
